# Association between the IL-10 and IL-6 polymorphisms and brucellosis susceptibility: a meta-analysis

**DOI:** 10.1186/s12881-020-01006-0

**Published:** 2020-03-30

**Authors:** Xiaochun Jin, Yueyuan Wu, Shuzhou Yin, Xu Chen, Youtao Zhang

**Affiliations:** 1 Department of Anesthesiology, Suzhou Kowloon Hospital, Shanghai Jiaotong University School of Medcine, Suzhou, 215028 People’s Republic of China; 2 Department of Clinical Laboratory, First Affiliated Hospital of Soochow University, 188 Shizi Road, Suzhou, 215006 People’s Republic of China

**Keywords:** Brucellosis, Interleukin-10, Interleukin-6, Polymorphism, Meta-analysis

## Abstract

**Background:**

Brucellosis is a quite normal zoonotic infection, which is caused by immediate contact with animals infected with Brucella or its products. IL-10 (− 1082 G/A, − 819 C/T, − 592C/A) and IL-6 -174 G/C polymorphisms have a great relationship with IL-10 and IL-6 production, which brings about Brucellosis pathogenesis and development. So far, the results of published literatures were controversial. Now, we perform a meta-analysis in different ethnic populations to get a more precise estimate of above polymorphisms with Brucellosis susceptibility.

**Methods:**

Both OR and corresponding 95%CI were enrolled to make an assessment of the association strength through extracting genotyping frequency of cases and controls. The χ2-test based Q-statistic and I^2^ statistics were applied. If there was no evident heterogeneity, the fixed-effects model would be applied. If not, the random-effects model would be used.

**Results:**

The significant associations were only found in Asian population of − 819 loci under three genetic models as follows: (Allele model: OR = 0.60, 95%CI = 0.44–0.82, *P* = 0.001), (homozygote comparison: OR = 0.24, 95%CI = 0.09–0.62, *P* = 0.003), (recessive genetic model: OR = 0.22, 95%CI = 0.05–0.91, *P* = 0.036).

**Conclusion:**

In conclusion, IL-10 − 819 loci polymorphism contributes no risk to Caucasian population but may be associated with decreased risk in Asian population. And IL-10 -1082 G/A, 592 loci and IL-6 -174 G/C polymorphism are not associated with Brucellosis risk.

## Background

Brucellosis is a quite normal zoonotic infection, which is caused by immediate contact with animals infected with Brucella or its products. Although the number of Brucellosis patients is relatively small, it remains a severe problem and it is very popular locally in most areas of Asia and Africa [1, 2]. Some patients show onset fever, some show fatigue or sweating. Multiple clinical manifestations can be shown by Brucellosis. What’s worse, nontypical clinical presentation brings difficulties to diagnosis. The exact pathogenesis of Brucellosis remains unknown. It has been reported that cell-mediated immunity is considered to play a crucial role in immunity response to Brucellosis infection [3]. CD4^+^ and CD8^+^ T lymphocytes are considered as playing a key role in cellular immunity as they can release IFN-λ and activate the functions in macrophages [4, 5]. Additionally, Interleukin-10 (IL-10) is a crucial cytokine contributing to resist inflammation, which makes various biological effects on multiple types of cell. After the infection of Brucella pathogen, IL-10 can lead to the production drawdown of IFN-λ and inhibition of macrophages function [6].

IL-6 is another cytokine, not only involving in the process of inflammation response but also regulating multiple biological processes such as metabolism, regeneration and nervous system [7]. It has been demonstrated that polymorphism can influence the expression of cytokine and play a crucial role in infectious diseases [8, 9].

There are quite a few literatures demonstrating that hereditary factors play crucial parts in the development of Brucellosis [10–14]. Apart from IL-6 -174 G/C polymorphism, a large number of documents have focused on IL-10 promoter polymorphisms, including position − 1082(G → A) (rs1800896), − 819(C → T) (rs1800871) and − 592(C → A) (rs1800872). However, inconsistent results were obtained. In the present study, we carry out a meta-analysis to obtain a more precise estimate of the above polymorphisms. In addition, the present study is a multi-ethnic study because ethnic populations may contribute tremendous influence to genetic polymorphisms of IL-10 and IL-6.

## Methods

### Search strategy

The present meta-analysis was performed on account of predefined protocol named Meta-analysis Of Observational Studies in Epidemiology (MOOSE) group [15]. Two databases consisting of PubMed and Embase were put into use by searching keywords as follows: (“IL” or “Interleukin”) and “Brucellosis” and “polymorphism”. The expiration date was the end of February, 2020. We reviewed titles and abstracts of all citations and retrieved studies. We did not limit anything of literatures such as its language, regional culture, publication year and sample size. After these studies were received, their corresponding references were also investigated for other possible studies.

### Inclusion and exclusion criteria

The inclusion criteria consisted of three items: (a) a case-control research or short communication; (b) assessment of relationship between IL polymorphisms and Brucellosis susceptibility; (c) providing enough data to judge the final result or the present data can predict the final result. Accordingly, other types of research were excluded such as case report, review article.

### Data extraction

The whole information and data were independently extracted by first author (Yueyuan Wu, Shuzhou Yin and Xiaochun Jin) and the final information was reviewed by final referees (Xu Chen and Youtao Zhang). The present study is a multi-ethnic study. Different ethnic descents were categorized as African, Asian, and Caucasian. To get a accurate result, three authors (Shuzhou Yin, Yueyuan Wu and Xiaochun Jin) were asked to check all data and information. If they could not reach an agreement, they would check the above data and information again and have a meeting trying to come to an agreement. If the controversial results still existed, the final referees (Xu Chen and Youtao Zhang) would be asked to give the final decision.

### Methodological quality assessment

Three authors (Shuzhou Yin, Yueyuan Wu and Xiaochun Jin) were asked to perform methodological quality evaluation according to predefined assessment criteria (Table 1), which was based on the items of Jiang et al. [16]. The scores with the range of 0(lowest) to 18(highest) were based on several assessment items consisting of credibility of controls, matching criteria, diagnostic criteria, genotyping examination, sample size and Hardy-Weinberg equilibrium. The above assessment items were performed based on popular epidemiological methods and Brucellosis special characteristic. Literatures whose scores less than 12 were considered as “low-quality” literatures. However, the literatures with scores equal to or more than 12 were regarded as “high-quality” studies.

**Table 1:** Detailed evaluation criteria focused on included studies

Criterion of evaluation	score
Reliability of controls	
Live in the area locally	3
Volunteers who conduct blood or organ transplant	2
Patients who is not related with Brucellosis	1
Not reported or cannot get the detailed information	0
Matching standard	
All including age, sex and ethnicity	3
Ethnicity only	1.5
Not reported or cannot get the detailed information	0
Diagnosis of Brucellosis	
According to clinical manifestation and high antibodies	3
According to history or other examination	1.5
Not reported or cannot get the detailed information	0
Genotyping methods	
by “blinded” status	3
Not reported or cannot get the detailed information	0
Hardy-Weinberg equilibrium	
The compliance is good	3
Not reported or cannot get the detailed information	0
Sample size	
More than 500	3
More than 200 and less than or equal to 500	2
More than 100 and less than or equal to 200	1
less than 100	0

### Statistical analysis

Both the OR and 95%CI were estimated to make the assessment of association power in four different genetic models. For position − 174 G/C of IL-6, there were allele comparison (G versus C), homozygote comparison (GG versus CC), recessive model (GG versus GC/CC), and dominant model (GG/GC versus CC). For position − 1082 G/A of IL-10, there were allele comparison (A versus G), homozygote comparison (AA versus GG), recessive model (AA versus AG/GG), and dominant model (AA/AG versus GG). For position − 819 C/T of IL-10, there were allele comparison (T versus C), homozygote comparison (TT versus CC), recessive model (TT versus TC/CC), and dominant model (TT/TC versus CC). For position − 592 loci of IL-10, there were allele comparison (A versus C), homozygote comparison (AA versus CC), recessive model (AA versus AC/CC), and dominant model (AA/AC versus CC). The χ2-test based Q-statistic and I^2^ statistics were used. If there was no evident heterogeneity, the fixed-effects model would be applied [17]. If not, the random-effects model would be used [18]. Sensitivity analysis was used to evaluate the stability of the results. Funnel plots and Egger’s test were applied to detect the potential publication bias [19]. All statistics were conducted using Stata software (version 12.0; StataCorp LP, College Station, TX, USA).

## Results

### Eligible studies

**Fig. 1:**
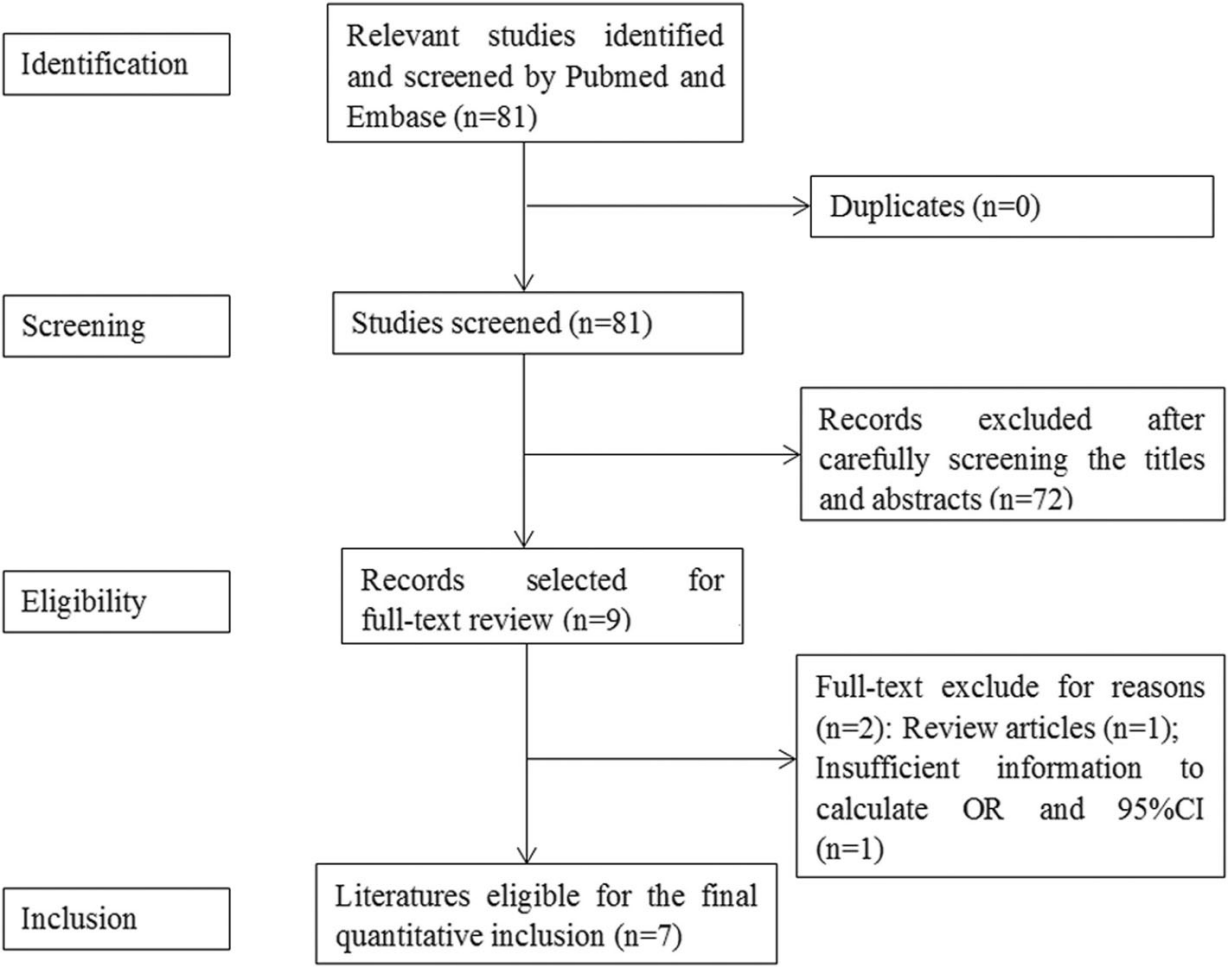
Flow diagram for identification of eligible studies for this meta-analysis

A flow chart of the search course is displayed in Fig. 1. Seven literatures were enrolled in our meta-analysis [10, 20–25]. The detailed information of seven studies was listed in Table 2.

**Table 2:** Characteristics of studies included in this meta-analysis

Literature	Country (Ethnics)	Genotyping methods	Source of control	Sample size (Case/Control)	Studied polymorphism	conformity of HWE	Quality score
Bravo(2003) [10, 12]	Caucasian (Spain)	PCR-SSP	PB	83/101	IL-10(−1082,-819,−592)	Yes	14
Budak(2007) [20]	Caucasian (Turkey)	PCR-SSP	PB	40/50	IL-10(−1082,-819,-592); IL-6(−174)	Yes	12
Bravo(2008) [25]	Caucasian (Spain)	PCR-SSP	PB	82/102	IL-6(−174)	Yes	13
Rasouli(2008) [22]	Asian (Iran)	PCR-RFLP	PB	190/81	IL-10(−1082,-819,-592)	Yes	13
Karaoglan(2009) [21]	Caucasian (Turkey)	PCR-SSP	PB	85/85	IL-10(−1082,-819); IL-6(−174)	Yes	13
Asaei(2013) [24]	Asian (Iran)	PCR-RFLP	PB	196/82	IL-6(−174)	Yes	14
Kazemi(2016) [23]	Asian (Iran)	PCR-RFLP	PB	60/60	IL-10(−1082,-819,-592); IL-6(−174)	Yes	13

*PB* Population–based, *PCR-SSP* Polymerase chain reaction-sequence-specific primer, *WE* Hardy–Weinberg equilibrium in control population, *PCR–RFLP* Polymerase chain reaction-restriction fragment length polymorphism.

### Quantitative synthesis of data

**Fig. 2:**
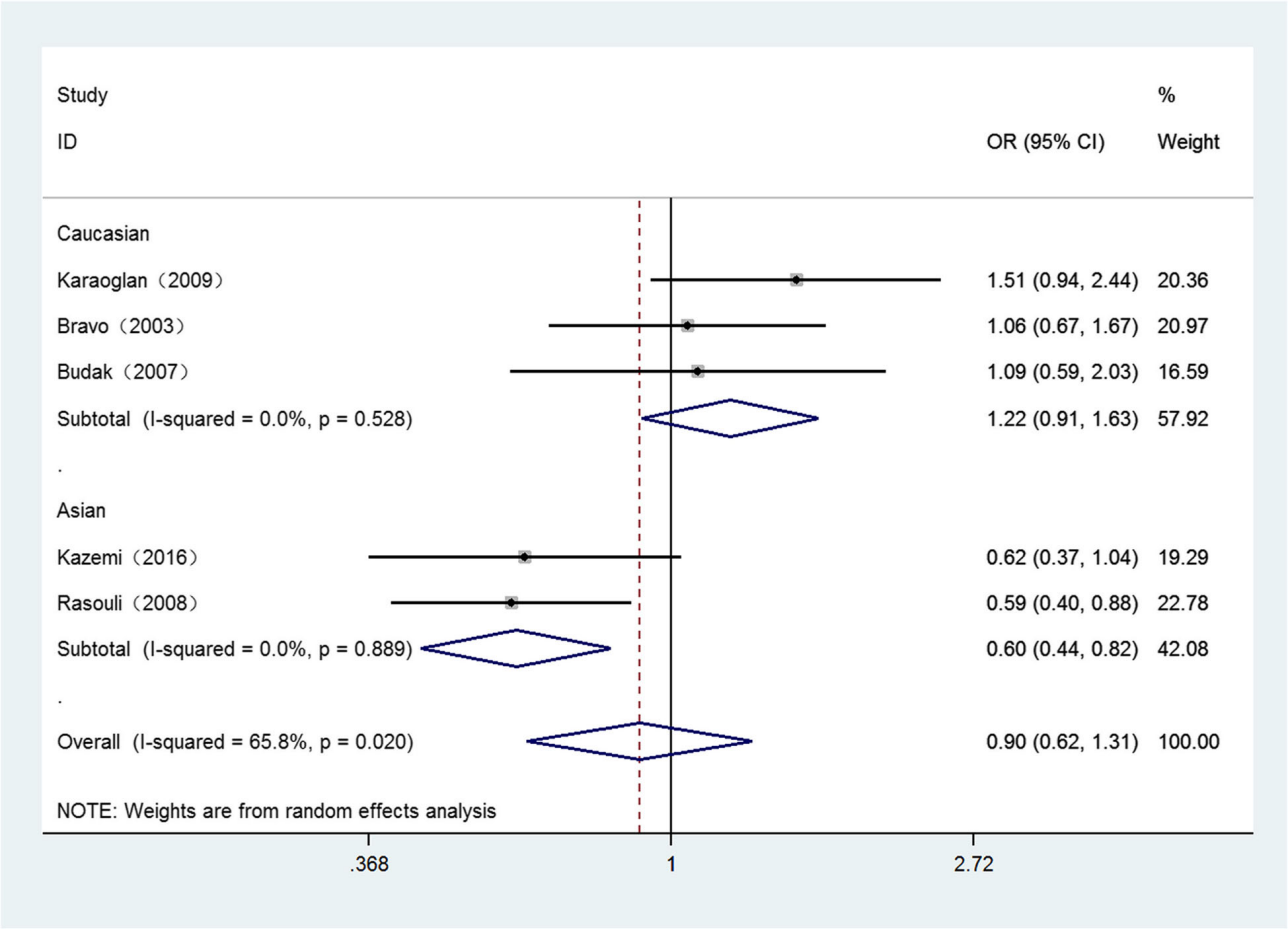
Forest plot of IL-10 -819 C/T polymorphism on Brucellosis risk in allele model (T vs. C)

**Fig. 3:**
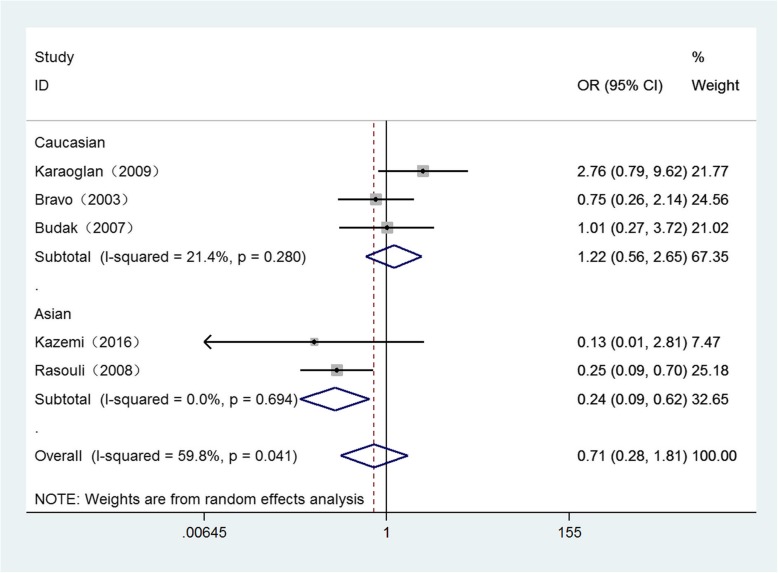
Forest plot of IL-10 -819 C/T polymorphism on Brucellosis risk in homozygote model (TT vs. CC)

**Fig. 4:**
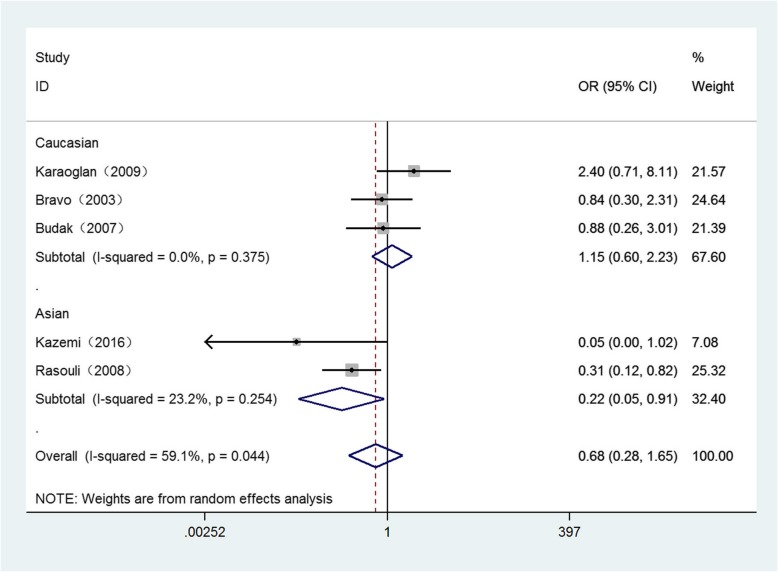
Forest plot of IL-10 -819 C/T polymorphism on Brucellosis risk in recessive model (TT vs. CC/TC)

Detailed results for the relationship between these polymorphisms and Brucellosis susceptibility are displayed in Table 3. *P* < 0.05 is considered as a significant association. Overall, significant associations were only found in Asian population of − 819 loci under three genetic models as follows: (Allele model: OR = 0.60, 95%CI = 0.44–0.82, *P* = 0.001) (Fig. 2), (homozygote comparison: OR = 0.24, 95%CI = 0.09–0.62, *P* = 0.003) (Fig. 3), (recessive genetic model: OR = 0.22, 95%CI = 0.05–0.91, *P* = 0.036) (Fig. 4).

**Table 3:** The general results for the association between IL-10, IL-6 polymorphisms with Brucellosis risk

Comparison	Group (sample size)	Test of association			Mode	Test of Heterogeneity		
		OR	95%CI	P		x^2^	P	I^2^
IL-6-174 (G → C)								
G versus. C	Overall(452/377)	1.09	0.87–1.36	0.468	Fixed	1.27	0.867	0
	Caucasian(202/235)	1.09	0.82–1.46	0.544	Fixed	1.14	0.567	0
	Asian(250/142)	1.07	0.76–1.52	0.685	Fixed	0.13	0.724	0
GG versus. CC	Overall(452/377)	1.28	0.71–2.28	0.410	Fixed	4.33	0.363	7.7
	Caucasian(202/235)	1.15	0.43–3.12	0.780	Fixed	3.61	0.164	44.6
	Asian(250/142)	1.78	0.69–4.55	0.232	Fixed	0	0.976	0
GG versus. CC/GC	Overall(452/377)	1.03	0.77–1.38	0.852	Fixed	3.64	0.457	0
	Caucasian(202/235)	1.04	0.63–1.73	0.869	Fixed	3.37	0.185	40.7
	Asian(250/142)	0.97	0.62–1.52	0.905	Fixed	0.15	0.699	0
GG/GC versus. CC	Overall(452/377)	1.30	0.62–2.73	0.487	Fixed	6.95	0.138	42.5
	Caucasian(202/235)	1.16	0.35–3.87	0.809	Random	5.53	0.063	63.8
	Asian(250/142)	1.87	0.74–4.71	0.184	Fixed	0.02	0.891	0
IL-10-1082 (G → A)								
A versus. G	Overall(458/377)	0.82	0.62–1.08	0.152	Fixed	6.94	0.139	42.4
	Caucasian(208/236)	0.76	0.49–1.18	0.217	Random	4.90	0.086	59.1
	Asian(250/141)	0.89	0.58–1.37	0.602	Fixed	1.78	0.182	44.0
AA versus. GG	Overall(458/377)	1.51	0.87–2.60	0.142	Fixed	5.07	0.281	21.1
	Caucasian(208/236)	1.36	0.58–3.18	0.484	Random	4.20	0.123	52.4
	Asian(250/141)	1.95	0.83–4.58	0.124	Fixed	0.37	0.544	0
AA versus. GG/GA	Overall(458/377)	0.80	0.49–1.30	0.360	Random	9.76	0.045	59
	Caucasian(208/236)	0.62	0.39–0.98	0.043	Fixed	2.75	0.252	27.4
	Asian(250/141)	1.42	0.39–5.23	0.598	Random	4.66	0.031	78.5
AA/GA versus. GG	Overall(458/377)	0.72	0.39–1.32	0.291	Fixed	7.06	0.133	43.4
	Caucasian(208/236)	0.96	0.44–2.09	0.916	Random	2.75	0.126	51.7
	Asian(250/141)	0.42	0.19–0.94	0.035	Fixed	4.66	0.678	0
-819 (C → T)								
T versus. C	Overall(458/377)	0.90	0.62–1.31	0.587	Random	11.71	0.02	65.8
	Caucasian(208/236)	1.22	0.91–1.63	0.190	Fixed	1.28	0.528	0
	Asian(250/141)	0.60	0.44–0.82	0.001	Fixed	0.02	0.889	0
TT versus. CC	Overall(458/377)	0.71	0.28–1.81	0.471	Random	9.96	0.041	59.8
	Caucasian(208/236)	1.22	0.56–2.65	0.615	Fixed	2.54	0.280	21.4
	Asian(250/141)	0.24	0.09–0.62	0.003	Fixed	0.15	0.694	0
TT versus. CC/TC	Overall(458/377)	0.68	0.28–1.65	0.393	Random	9.77	0.044	59.1
	Caucasian(208/236)	1.15	0.60–2.23	0.670	Fixed	1.96	0.375	0
	Asian(250/141)	0.22	0.05–0.91	0.036	Fixed	1.30	0.254	23.2
TT/TC versus. CC	Overall(458/377)	1.33	0.73–2.44	0.353	Random	15.88	0.003	74.8
	Caucasian(208/236)	1.31	0.90–1.90	0.160	Fixed	0.44	0.802	0
	Asian(250/141)	1.62	0.19–13.80	0.658	Random	13.99	0	92.9
-592 (C → A)								
A versus. C	Overall(373/292)	0.90	0.64–1.26	1.524	Fixed	7.56	0.109	47.1
	Caucasian(123/151)	1.05	0.70–1.56	0.813	Fixed	2.21	0.331	9.6
	Asian(250/141)	0.78	0.44–1.38	0.393	Random	3.55	0.059	71.9
AA versus. CC	Overall(373/292)	0.58	0.32–1.06	0.076	Fixed	4.49	0.344	10.9
	Caucasian(123/151)	0.77	0.32–1.85	0.564	Fixed	0.76	0.683	0
	Asian(250/141)	0.48	0.14–1.67	0.248	Random	2.99	0.084	66.5
AA versus. CC/CA	Overall(373/292)	0.63	0.37–1.07	0.086	Fixed	2.91	0.573	0
	Caucasian(123/151)	0.86	0.38–1.94	0.710	Fixed	0.05	0.167	0
	Asian(250/141)	0.51	0.19–1.33	0.168	Random	1.91	0.078	47.7
AA/CA versus. CC	Overall(373/292)	0.80	0.51–1.26	0.345	Fixed	6.10	0.192	34.4
	Caucasian(123/151)	0.78	0.34–1.80	0.567	Fixed	3.89	0.235	30.9
	Asian(250/141)	0.80	0.40–1.67	0.535	Random	3.15	0.076	68.2

### Sensitivity analysis and publication Bias

Sensitivity Analysis was carried out to indicate single literature’s effect on the final result through continuous removal of individual studies under every genetic model. In the present study, our results could not be affected by any study, suggesting its reliability and robustness. Although slightly asymmetrical funnel plots were discovered (*P* = 0.806), we could not find any distinct publication bias by Egger’s test under allele model (*P* = 0.509) or recessive model (*P* = 0.509).

## Discussion

Previous studies have explored the association of IL-10 and IL-6 polymorphisms with Brucellosis risk. The present meta-analysis consists of 7 studies for IL-10 and IL-6 polymorphisms. To the best of our knowledge, the present research was the first to investigate the relationship between IL-10 and IL-6 polymorphisms and Brucellosis risk. Our meta-analysis shows that IL-10 -819 loci polymorphism contributes no risk to Caucasian population but may be associated with decreased risk in Asian population. And other polymorphisms of IL-10 and IL-6 are not related with Brucellosis susceptibility. For IL-6 -174 G/C polymorphism, Budak et al. reported in 2007 that GG genotype was more common in patients of Brucellosis than healthy controls (40 cases and 50 controls) and concluded that GG genotype was a risk factor in the development of Brucellosis [20]. However, the other four literatures reported no association between IL-6 -174 G/C polymorphism and Brucellosis susceptibility. We consider sample size as an important factor contributing to the above discrepancy. Small-study bias is not an emerging event when performing genetic association studies [26]. The current result of IL-10 -819 loci polymorphism may be attributed to race. It is well-known that different races have discrepant genotype number and allele frequency. So that it is necessary to conduct subgroup analysis by race. However, we made a conservative conclusion. Only two literatures with 250 cases and 141 controls were enrolled in our study. Considering the small sample size of eligible studies, a new updated study needs to be urgently conducted. Regarding the significant association found at IL10–1082 AA genotyping in Caucasian, we feel that it is likely a false positive, which is based on two reasons. Firstly, the *P* value approximates 0.05 and is a critical value. Secondly, the *P* values of other genetic models are 0.217, 0.484, and 0.916(*P* > 0.05).

Extensive variations have been established in the frequencies of cytokine polymorphism among different healthy population, including the − 1082 G/A polymorphism, which has been investigated most widely in healthy populations. The allele frequency show wide difference in different countries and regions. For example, the prevalence rate of IL-10 -1082 G allele was reported to be 42.5, 48.9 3.8 and 13.0% in Iranians, Norwegians, Japanese and Koreans, respectively [27–30].

HWE has been demonstrated to be an important genetic equilibrium law. And if the genotype distribution of control population is not conforming to HWE law, selection bias would occur. It has been reported that several factors would contribute to HWE deviation including fixed mating, or allele do not achieve equilibrium condition and other possible factors, which may lead to inaccurate results of meta-analysis. In our meta-analysis, the problem contributes little influence on our results. Because genotype distributions of all studies are consistent with HWE, which improves reliability of our results greatly.

Genome-wide association study (GWAS) is undoubtedly an effective method to find the sequence of gene mutations, accordingly screening the specific SNP associated with disease. GWAS opens the door to the study of complex diseases by comparing patients’ SNP sites with control groups in whole genome, which identifies all variant allele mutation frequencies. It is regrettable that there is rare GWAS on Brucellosis. Sankarasubramanian etal pulished a study titled “Identification of genetic variants of Brucella spp. through genome-wide association studies” and they found special SNPs, which were closely related with human’s specificity and virulence. They also thought that origin of these special SNPs was early and might derive from *B. abortus* evolution [31]. However, we think this is just a new start in exploring species-specific SNPs of Brucellosis. In the future, we should investigate further in the genomes and their roles of Brucellosis.

Despite a lot of work we have done, there are still disadvantages existing, which should be remarkable here. Firstly, the present research is still a small-sample study and we should be careful in the results. Secondly, some miscellaneous factors including age, sex and other environment factors are not considered and calculated for final estimates. So that the results of our meta-analysis are on the account of unadjusted estimates. We should also be careful in the results.

## Conclusion

In conclusion, this is the first meta-analysis which investigates the association between IL-10 polymorphism and Brucellosis risk. In conclusion, IL-10 -819 loci polymorphism contributes no risk to Caucasian population but may be associated with decreased risk in Asian population. And IL-10 -1082 G/A, 592 loci and IL-6 -174 G/C polymorphism are not associated with Brucellosis risk.

## Data Availability

All data generated or analysed during this study are included in this published article and its supplementary information files.

## Abbreviations

IL-10: Interleukin-10
IL-6: Interleukin-6
PB: Population-based
HWE: Hardy-Weinberg equilibrium
RFLP: Restricted Fragment Length Polymorphism
PM: Pneumococcal meningitis
MM: Meningococcal meningitis
CI: Confidence interval
OR: Odds ratio
SNP: Single nucleotide polymorphism
